# Clinical utility and methodological implementation of smartphone-based mobility analysis: a scoping review

**DOI:** 10.1007/s00415-026-14131-2

**Published:** 2026-09-15

**Authors:** Edwin H. Y Lui, Ralph Jasper Mobbs

**Affiliations:** 1 Faculty of Medicine, University of New South Wales, Sydney, NSW 2052 Australia; 2 NeuroSpine Surgery Research Group, Sydney, NSW 2031 Australia; 3 NeuroSpine Clinic, Prince of Wales Private Hospital, Randwick, NSW 2031 Australia; 4 Wearables and Gait Research Group (WAGAR), Prince of Wales Private Hospital, Randwick, NSW 2031 Australia

**Keywords:** Neurology, Smartphones, mHealth, Gait analysis, Mobility, Clinical utility

## Abstract

**Background:**

Mobility is an informative marker of neurological function, physiological reserve, and functional independence. Laboratory gait analysis requires costly specialized equipment, while research wearables can impose sustained-use burdens. Smartphone inertial measurement units (IMUs) offer a widely available platform for longitudinal assessment within a patient’s natural environment.

**Methods:**

Following PRISMA-ScR guidelines, PubMed, Embase, and Scopus were searched for peer-reviewed studies using internal smartphone sensors to map reported clinical applications, methodological implementation, validation approaches, and translational barriers.

**Results:**

Eighty-four studies met the inclusion criteria. Neurology was the most frequently represented clinical domain (36.9%), with additional applications in geriatrics (14.3%) and orthopedics (13.1%). 10.7% (*n*=9) were classified as validation-focused; the remainder had diagnostic (36.9%), prognostic (38.1%), or longitudinal monitoring (14.3%) aims. Most assessments remained controlled (54.8%), supervised (67.9%), and dependent on active testing protocol (73.8%), whereas only 23.8% captured passive, free-living mobility. Implementation was heterogeneous: 71.4% of protocols required fixed device placements, 82.1% used custom software, and 7.1% integrated with native platforms. The extracted measures included activity volume, gait-quality metrics, and machine-learning features derived from raw inertial data.

**Conclusion:**

Smartphones provide a widely available platform for multidimensional mobility sensing, but the mapped evidence largely concerns feasibility, technical validation, or associations with clinical status and outcomes. Routine clinical utility has not been established. Translation will require standardized acquisition and reporting, cross-device and cross-platform validation, context-aware analysis, privacy-preserving data governance, and prospective evidence that implementation improves clinical decisions or patient-relevant outcomes. Passive monitoring may complement standardized assessment by providing longitudinal measures of real-world performance.

**Supplementary Information:**

The online version contains supplementary material available at https://doi.org/10.1007/s00415-026-14131-2.

## Introduction

Mobility is an informative marker of neurological function, physiological reserve, and functional independence across the lifespan [1, 2]. Walking speed and gait characteristics have been associated with cognitive decline, mortality, neurodegenerative trajectories, and disease progression [3–5], while gait alterations may provide early indications of several neurological and musculoskeletal conditions [6, 7]. Despite this clinical relevance, assessments commonly rely on patient-reported outcome measures (PROMs) or periodic clinical visits [8]. These episodic assessments have limitations in representing a patient’s functional performance and mobility during daily life [9–11].

Dedicated wearables and specialized gait laboratories can provide objective functional data for conditions such as Parkinson disease and multiple sclerosis, but their use in longitudinal daily-life monitoring remains limited [12]. Reference gait analysis systems require laboratory equipment, such as three-dimensional optoelectronic motion capture and force plates, that is not suitable for routine continuous monitoring [13, 14]. Specialized research wearables may also impose costs and sustained-use burdens and may not fully capture spontaneous behavior in daily life [15, 16].

Smartphones offer a potentially scalable complement to these approaches [17]. Their built-in inertial measurement units (IMUs), widespread ownership, and routine presence in daily life may support longitudinal mobility assessment with less additional equipment. Smartphone sensing could therefore complement episodic clinical assessment with objective measures collected within a patient’s natural environment [18].

Therefore, this scoping review aims to map the reported clinical applications, validation approaches, prognostic potential, methodological implementation, and translational barriers of smartphone-based mobility assessment.

## Methods

### Study design and eligibility criteria

This scoping review was conducted in accordance with the PRISMA-ScR (Preferred Reporting Items for Systematic Reviews and Meta-Analyses extension for Scoping Reviews) guidelines to systematically map the literature evaluating internal smartphone IMUs for clinical mobility analysis [19]. A comprehensive search across PubMed, Embase, and Scopus was finalized on April 30, 2026, targeting three core concepts: smartphones, mobility, and clinical utility.

Eligibility was defined using the PRISMA-ScR PCC (Population, Concept, Context) framework. The population included healthy adults and patients with clinical pathologies across all medical specialties. The concept focused on utilizing internal smartphone IMUs to objectively quantify mobility, gait, and postural transitions. The context encompassed all operational environments, from controlled laboratories to unconstrained, free-living community settings.

Eligible studies were restricted to peer-reviewed, English-language articles presenting original human mobility data derived from consumer smartphone IMUs. Studies were excluded if they relied on external wearables, non-kinematic tracking such as GPS, cameras, or subjective surveys lacking inertial sensor data.

### Data extraction and charting

A standardized data extraction framework was developed to capture the methodological, technological, and clinical dimensions of each included study. Data were systematically charted into four overarching domains to map the translational gap between laboratory-based mobility research and real-world implementation: (1) Clinical targets and purpose, categorized by medical specialty, pathology, and primary study purpose as validation, diagnosis, prognostication, or longitudinal monitoring; (2) Methodological implementation, categorized by operational context, level of patient engagement, and task complexity; (3) Technological infrastructure, evaluated by physical smartphone placement and software architecture; and (4) Extracted metrics and validation standards, categorized by the mobility measures reported and the reference methods used to assess technical or concurrent validity.

### Data synthesis

Given the scoping nature of this review and the substantial heterogeneity in study designs, sensor placements, outcomes, and algorithmic processing, quantitative meta-analysis was not appropriate. Instead, a narrative synthesis supported by descriptive statistics was performed. As the review was designed to map the breadth and characteristics of the literature rather than determine the certainty of evidence, no formal risk of bias or methodological quality appraisal was undertaken. The heterogeneity of the included studies and absence of a common benchmarking framework also limited direct comparisons of methodological quality across studies. Accordingly, study counts were interpreted as indicators of the distribution of research activity rather than the strength or certainty of evidence. This mapping enabled the identification of methodological bottlenecks, technological limitations, and broader trends in research on smartphone-derived gait informatics.

## Results

**Fig. 1:**
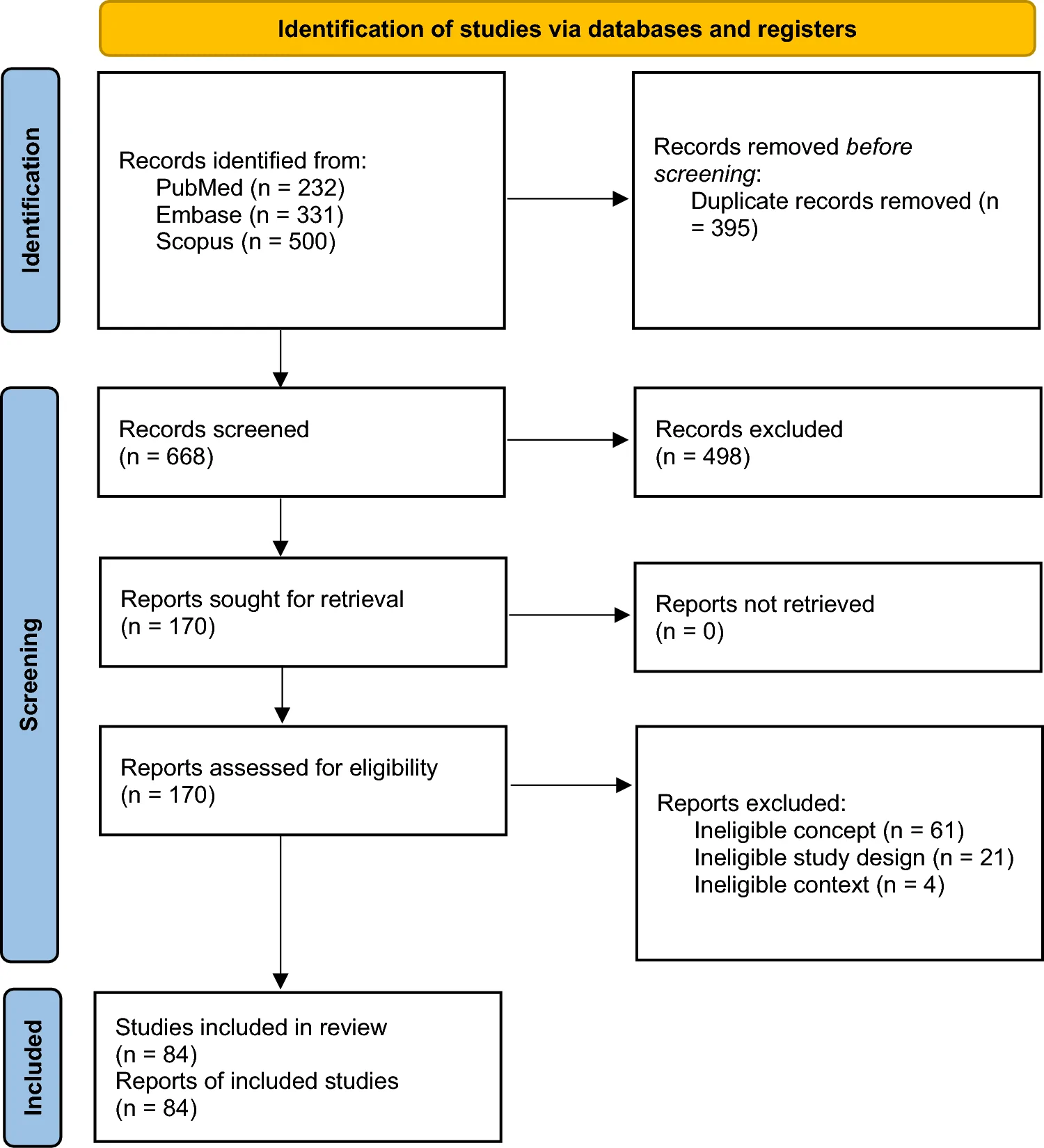
PRISMA flow diagram

From an initial yield of 1063 records, 84 peer-reviewed studies met the final inclusion criteria [20–103] (Supplementary File). Figure 1 illustrates the process of identifying and including articles, following the PRISMA-ScR guidelines.

### Clinical targets and applications

**Fig. 2:**
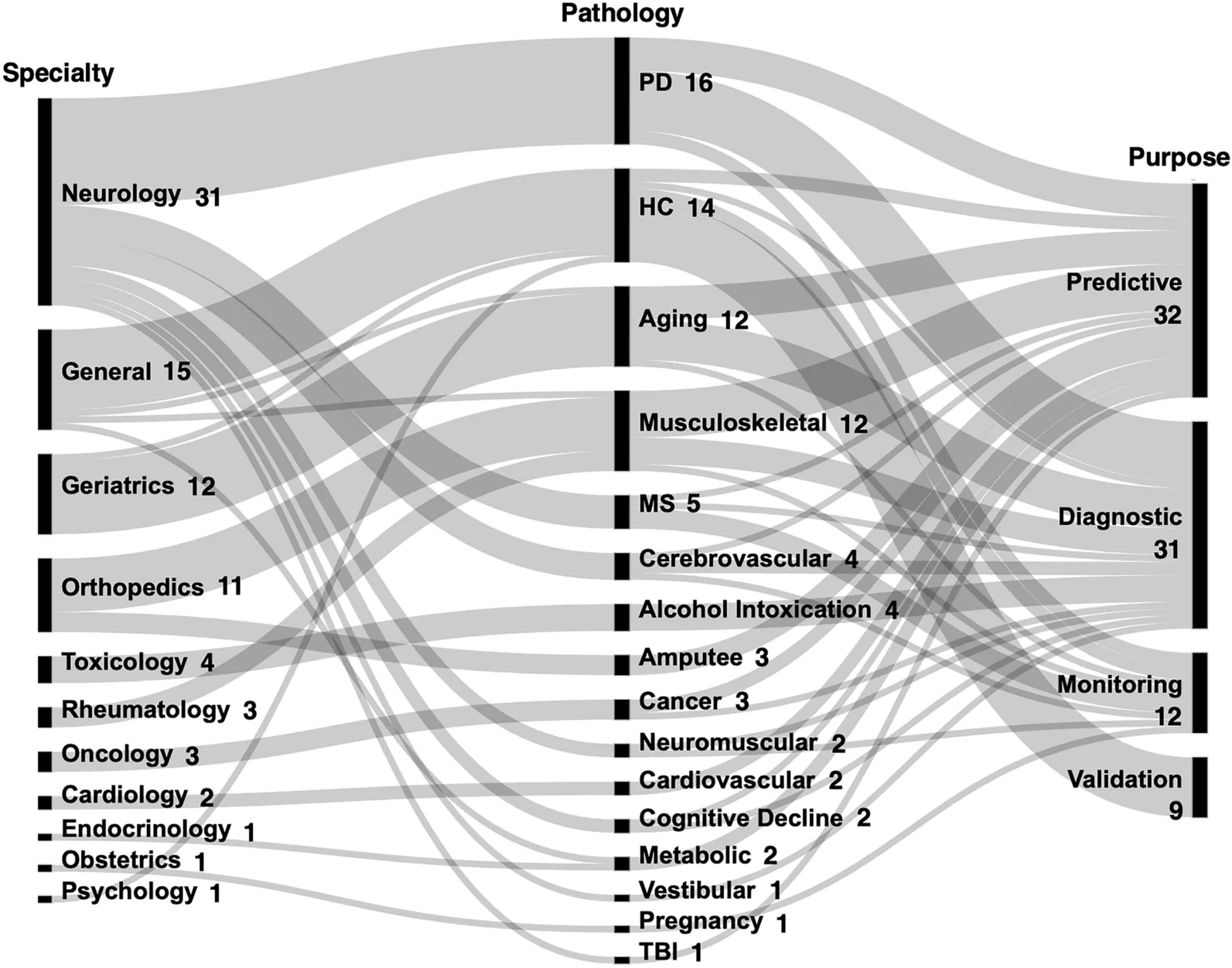
Distribution of studies across specialties, pathologies, purpose. *HC* healthy controls, *MS* multiple sclerosis, *PD* Parkinson disease, *TBI* traumatic brain injury

Smartphone-based mobility analysis has been investigated across multiple medical specialties and populations (Fig. 2). Neurology was the most frequently represented domain (36.9%, *n*=31), particularly Parkinson’s disease (19.0%, *n*=16) and multiple sclerosis (6.0%, *n*=5). Orthopedic studies involving the spine, hip, knee, and ankle accounted for 13.1% (*n*=11) of studies, while geriatric populations accounted for 14.3% (*n*=12). Rheumatology and oncology each accounted for 3.6% (*n*=3), while healthy-control cohorts were included in 16.7% (*n*=14), principally in feasibility and validation studies. Only 10.7% (*n*=9) were classified with validation as their primary purpose. The remaining 89.3% (*n*=75) had clinically directed aims: prognostication (38.1%, *n*=32), diagnosis (36.9%, *n*=31), or longitudinal monitoring (14.3%, *n*=12). These studies commonly examined relationships between smartphone-derived mobility measures and clinically relevant states or outcomes, including disease status, functional recovery, surgical complications, and post-discharge fall risk.

### Methodological implementation

**Fig. 3:**
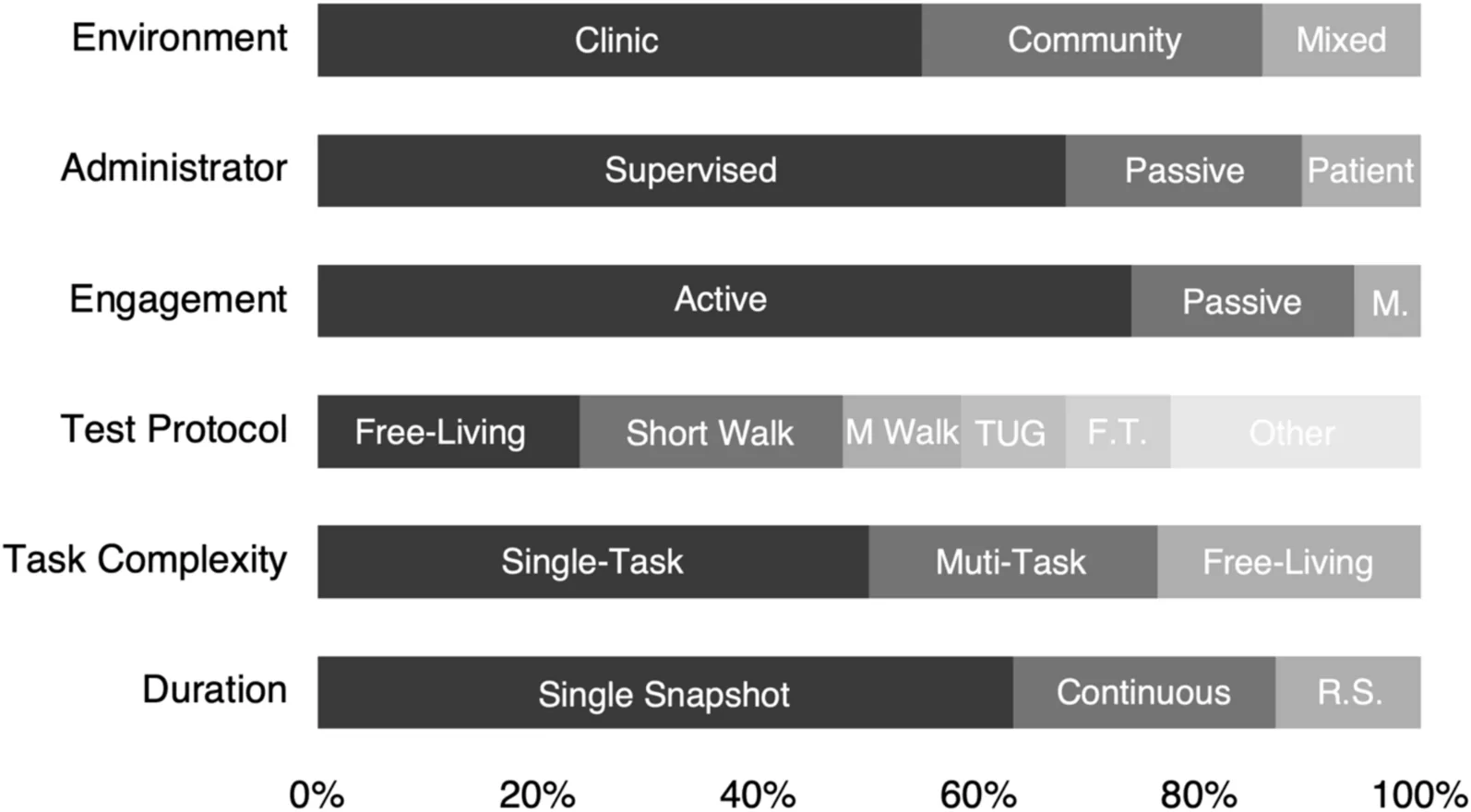
Proportional distribution of assessment methodologies extracted from the literature review. Data are categorized across six operational domains: environment, administrator, engagement, test protocol, task complexity, and duration. *M* mixed, *M Walk* medium walk, *TUG* timed up and go, *F.T*. functional test, *R.S*. repeated snapshot

Methodological implementation was concentrated in controlled, supervised, and active assessment frameworks (Fig. 3). Clinical or laboratory environments accounted for 54.8% (*n*=46) of studies, while 31.0% (*n*=26) collected data in community or free-living home settings. Assessments were supervised by clinicians or researchers in 67.9% (*n*=57) and required active participant engagement in 73.8% (*n*=62). Structured evaluations, including short- or medium-distance walk tests, the TUG, and the 6MWT, were used in 76.2% (*n*=64).

Task complexity was most commonly restricted to single-task walking (50.0%, *n*=42), while 26.2% (*n*=22) incorporated multiple tasks or cognitive distraction to evaluate mobility under divided attention. Passive, unstructured monitoring in free-living conditions was used in 23.8% (*n*=20) of studies. Consistent with the predominance of active assessment, 63.1% (*n*=53) of studies collected a single cross-sectional snapshot, whereas continuous longitudinal monitoring was reported in 23.8% (*n*=20).

### Placement and software

**Fig. 4:**
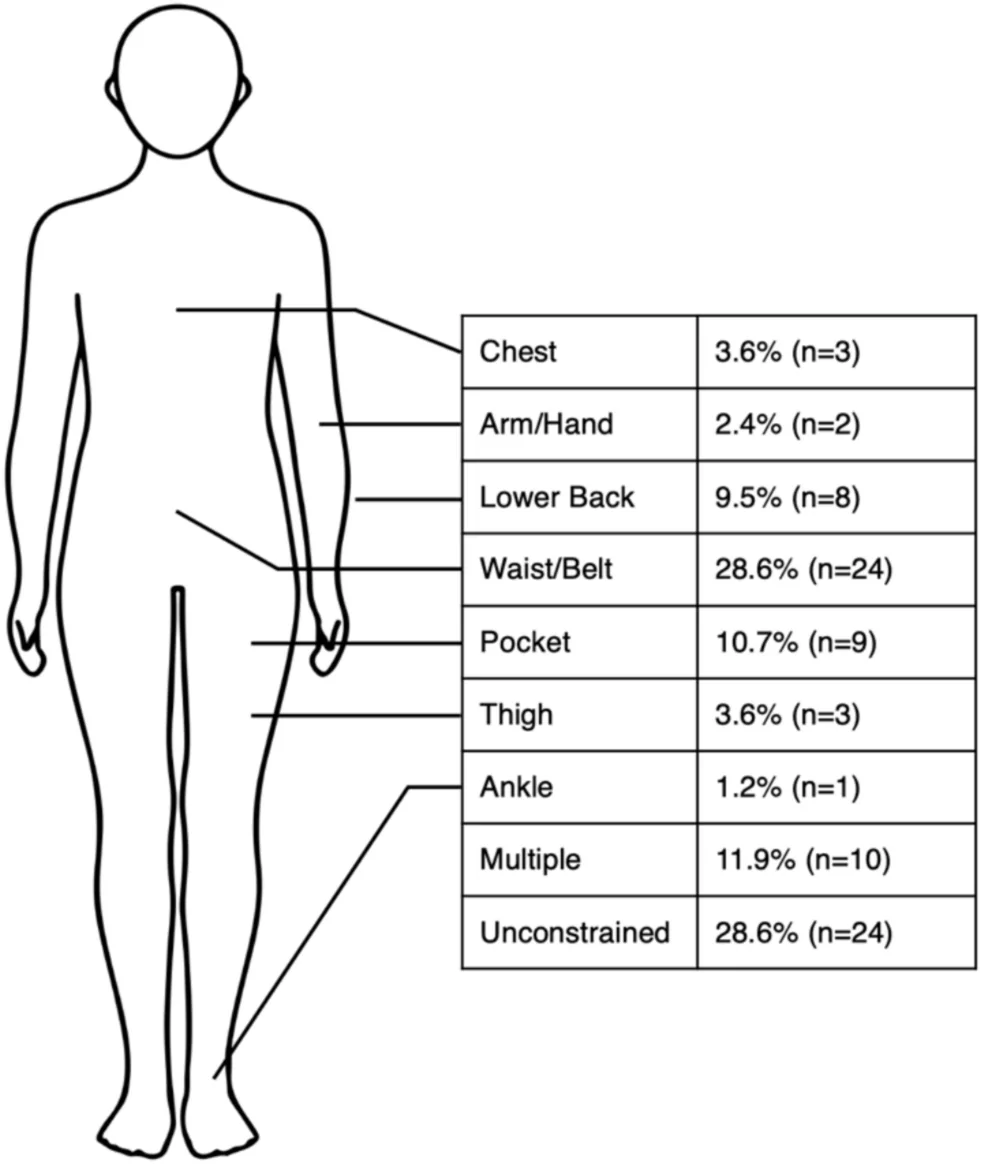
Reported anatomical locations for smartphone device placement. The schematic illustrates the specific physical sites where sensors were affixed during the assessment protocols

Fixed anatomical smartphone placement was required in 71.4% (*n*=60) of studies (Fig. 4). Belts, straps, or pouches were used to secure devices at the waist (28.6%, *n*=24), lower back (9.5%, *n*=8), or other anatomical landmarks. Naturalistic, unconstrained placement was used in 28.6% (*n*=24), and 11.9% (*n*=10) evaluated multiple placements to compare signal reliability across body sites.

Software architectures varied across studies. Custom-built applications or proprietary research platforms (e.g., mPower, GaitTrack, and GaitMate) were reported in 82.1% (*n*=69), commercial off-the-shelf applications (e.g., mymobility, OneStep, and Kinetikos) in 15.5% (*n*=13). Only 7.1% (*n*=6) of the reviewed studies integrated data collection from native, operating-system-level health platforms, such as Apple HealthKit or Google Fit.

### Extracted metrics and validation standards

Studies reported a broad range of mobility measures and machine-learning features derived from smartphone inertial sensors. In 32.1% (*n*=27), raw triaxial acceleration was used to construct feature sets for classification rather than to report explicit kinematic parameters. Among studies reporting interpretable spatiotemporal or activity-volume measures, gait speed was the most frequent (38.1%, *n*=32), followed by step count (33.3%, *n*=28), cadence (32.1%, *n*=27), and step length (28.6%, *n*=24). Gait-quality measures were less frequently reported, including stance-to-swing phase ratios (17.9%, *n*=15), gait asymmetry indices (16.7%, *n*=14), temporal variability measures (9.5%, *n*=8), and nonlinear measures such as approximate entropy (8.3%, *n*=7). Validation approaches commonly included comparison with three-dimensional optoelectronic motion capture, pressure mats, dedicated clinical wearables, or validated clinical rating scales. Such comparisons primarily assess technical or concurrent validity and do not, by themselves, demonstrate clinical utility.

## Discussion

This mapping of 84 studies demonstrates broad interest in smartphone-based mobility sensing, but also shows that methodological implementation remains concentrated in controlled, supervised, and actively prompted assessment. By synthesizing evidence across neurology, orthopedics, geriatrics, and other specialties, this review identifies technological, methodological, and behavioral barriers to the development of interpretable longitudinal mobility measures.

### Clinical applications and translational maturity

Neurology was the predominant domain (36.9%, *n*=31), with additional applications in geriatrics (14.3%, *n*=12) and orthopedics (13.1%, *n*=11). This distribution indicates that smartphone-derived mobility measures are being investigated across multiple clinical populations; it does not by itself establish their validity or clinical utility. The underlying clinical relevance of mobility is supported by established associations between gait and health outcomes [1, 2], but the performance and utility of specific smartphone-derived measures require further evaluation.

Only 10.7% (*n*=9) of studies were classified with validation as their primary purpose, whereas 89.3% (*n*=75) investigated clinically directed diagnostic, prognostic, or longitudinal-monitoring applications. This distribution indicates that the field is not limited to testing sensor accuracy, many studies evaluate relationships between smartphone-derived mobility measures and clinical states, recovery trajectories, or future outcomes relevant to patient assessment and risk stratification.

However, technical validity, clinical validity, and clinical utility therefore require separate interpretation. Technical validity concerns whether a smartphone-derived measure is accurate and reliable relative to an appropriate reference method. Clinical validity concerns whether the measure is associated with, discriminates, or predicts a clinically meaningful state or outcome. Clinical utility requires evidence that using the measure changes clinical decisions, care pathways, or patient outcomes. The mapped literature demonstrates progress from validation-focused studies toward clinically directed evaluation, but routine implementation and downstream clinical benefit have not been established. Translation will require consensus definitions, standardized acquisition and reporting, multicenter external validation across devices, settings, and populations, prespecified actionable thresholds, and prospective evaluation of effects on clinical decisions and patient-relevant outcomes [18, 104].

### Ecological validity

Capturing mobility within a patient’s daily environment is a principal advantage of smartphone-based assessment because it measures functional performance beyond the clinic. Nevertheless, a methodological disconnect persists across the literature: 54.8% (n=46) of studies remain confined to controlled clinical or laboratory environments, and 73.8% (n=62) require active patient engagement. Supervised, structured protocols provide standardized estimates of physical capacity under observation, whereas free-living monitoring can additionally characterize habitual performance, fatigue, diurnal fluctuations, and longer-term variability that may not be observable during a brief clinic visit [9, 104]. These approaches are therefore complementary rather than interchangeable.

However, the scalability of smartphones does not itself ensure sustained participation. The mPower study enrolled more than 12,703 participants remotely but experienced substantial attrition, particularly in the healthy control cohort [17, 58, 73]. Although attrition is multifactorial, the burden of repeated active testing may have contributed. Brodke et al. demonstrated a lower-burden alternative by using routinely collected Apple Health data to examine longitudinal orthopedic recovery and pre-injury mobility [30]. Passive background architectures may therefore reduce participant burden and support more sustained data collection without requiring repeated prompted assessments.

Our synthesis suggests that passive, free-living monitoring is an important direction for scalable longitudinal assessment and for realizing the distinctive advantage of smartphone sensing. Only 23.8% (n=20) of the mapped studies currently capture unconstrained mobility within the patient’s natural environment. This approach should not replace standardized assessment entirely. Rather, periodic structured testing can provide an interpretable anchor for continuous measures of real-world performance.

However, the gain in ecological validity carries a potential trade-off in measurement standardization. Walking surface and gradient, footwear, assistive devices, concomitant activities, crowding, indoor or outdoor location, and urban or domestic context may influence derived mobility measures independently of clinical change. Variations in phone placement and orientation, as well as periods when the phone is not carried, may also add noise, cause misclassification, or introduce non-random missingness.

Importantly, contextual variation is not always measurement noise; it may reveal how environmental demands shape functional performance. The analytical objective should therefore be to distinguish context-related from health-related variation rather than eliminate all real-world variability. Approaches represented in the mapped literature include placement-robust models, automated detection and segmentation of walking bouts, steady-state filtering, and longitudinal within-person comparison [29, 30, 62, 65, 105]. Additional strategies include context detection or annotation, aggregation of repeated observations, comparison of equivalent walking bouts, and stratified or sensitivity analyses, while periodic standardized assessments can help distinguish changes in real-world performance from changes in physiological capacity [9, 104]. Although these approaches cannot eliminate contextual confounding, they may improve interpretability while preserving the lower-burden and ecological advantages of passive monitoring.

### Compliance with placement and platforms

The feasibility of longitudinal monitoring is influenced by sustained participant engagement, and our mapping identified several related technological barriers.

First, a significant physical implementation constraint remains: 71.4% (*n*=60) of the literature required fixed anatomical placements, such as the waist or lower back. This reproduces some of the burden associated with external wearables and may be impractical for older adults or people with cognitive impairment. Emerging research suggests that placement constraints may be mitigated through algorithms designed to accommodate variations in device position [29, 62, 65]. Background filtering can also identify periods of steady-state walking from data collected when the device is broadly coupled to the center of mass [105]. These approaches may support more naturalistic placements (28.6%, *n*=24), such as a pocket or bag, provided that algorithms are validated across placement and orientation conditions.

Beyond physical placement, variability in smartphone hardware and operating systems is an important source of measurement heterogeneity. The differences in accelerometer and gyroscope resolution, noise characteristics, sampling frequency, timestamp accuracy, calibration, and sensor-fusion pipelines can affect derived mobility metrics. Device and operating-system updates may further alter data acquisition during longitudinal studies, while power-management policies and background permissions can cause device-specific data loss during passive monitoring. These factors limit reproducibility and may reduce the transportability of algorithms developed using a single device model or platform. Future studies should report the device model, operating-system and application versions, sensor and sampling settings, calibration and preprocessing procedures, and missing-data handling. Cross-device and cross-platform validation against standardized reference protocols is also required before multicenter clinical implementation [61, 104].

Software fragmentation also persists as a barrier to clinical scalability. Custom-built applications or proprietary research platforms were used in 82.1% (*n*=69) of studies, contributing to isolated data and analytical pipelines. Native operating-system platforms such as Apple Health or Google Fit may offer a path toward greater interoperability and, where sufficient historical data are available, access to pre-event mobility estimates [105]. Recent studies in trauma and emergency medicine illustrate the feasibility of using these data to examine recovery trajectories and adverse-outcome risk [30, 32], but they do not establish that native platforms are standardized across devices or that their use improves clinical decisions. Such platforms should therefore be considered candidate infrastructure requiring transparent algorithms, cross-platform validation, and prospective evaluation.

### Mobility metrics and clinical interpretability

Macro-mobility measures, such as step count and distance, have been widely studied and can characterize activity volume, but provide limited information about gait quality. Gait-quality measures (26.2%, *n*=22), including stride-time variability, asymmetry, and double-support percentage, may provide complementary information about neurological and musculoskeletal function [106, 107]. However, their reliability, validity, and clinically meaningful interpretation must be established for each intended population, device placement, and monitoring context.

A key research challenge is the development of analytical and signal-processing methods that can derive interpretable mobility measures from unconstrained real-world data. Evaluation should extend beyond algorithm performance to reliability across contexts and clinically meaningful interpretation [104, 108].

### Privacy, data governance, and equity

Continuous passive mobility sensing introduces privacy and governance requirements beyond those of episodic assessment. Even without GPS, temporal and movement patterns may reveal daily routines, periods of inactivity, and changes in health or functional status. Data collection should therefore be proportionate to the intended purpose and supported by transparent consent that explains what is collected, how long it is retained, who can access it, whether it may be reused, and how data will be handled after withdrawal. Data minimization, on-device processing where feasible, encryption, role-based access, audit trails, and predefined retention and deletion policies should be incorporated by design. Governance must also address data ownership and portability, linkage with health records, third-party platform access, cross-jurisdictional storage, software or algorithm updates, and accountability for generated alerts or missed deterioration. Clinical implementation will additionally require institutional oversight and equitable alternatives for patients who do not own or consistently carry compatible smartphones [18, 104].

### Limitations

This review has several methodological limitations. Restricting eligibility to peer-reviewed, English-language articles excluded non-English-language studies and gray literature, including unpublished commercial validation studies. Heterogeneity in study design, clinical population, sensor placement, outcome definition, and algorithmic processing precluded quantitative meta-analysis and necessitated narrative synthesis. No formal risk of bias or methodological quality appraisal was performed. Consequently, the findings could not be weighted by study quality. Category frequencies should therefore be interpreted as distributions of research activity rather than evidence of clinical effectiveness, readiness, or certainty. Excluding external wearables, including smartwatches, allowed the review to focus on smartphone-based applications but may have omitted multi-device tracking ecosystems.

Device-level sensor specifications, operating-system versions, power-management settings, and background permissions were inconsistently reported, preventing systematic comparison of hardware and platform effects. Standardized technical reporting and common benchmarking frameworks are needed to improve reproducibility, although limitations reported in older studies may not reflect contemporary smartphones. Environmental and behavioral covariates were also inconsistently reported and were not systematically extracted. Their effects on free-living measures could not be quantified, limiting attribution of observed differences to clinical status alone.

## Conclusion

Smartphones are emerging as widely available platforms for multidimensional mobility sensing, and the predominance of diagnostic, prognostic, and monitoring studies over validation-focused work demonstrates movement toward clinically directed applications. Although routine clinical utility remains to be established, smartphone-derived mobility metrics represent promising candidate digital measures. Further translation will depend on standardized acquisition and reporting, cross-device and cross-platform validation, context-aware interpretation, privacy-preserving governance, actionable thresholds, and prospective evaluation of effects on clinical decisions and patient-relevant outcomes. With these foundations, passive monitoring could complement standardized assessment by providing longitudinal measures of real-world performance.

## Supplementary Information

Below is the link to the electronic supplementary material.

Supplementary file1 (DOCX 85 kb)

Supplementary file2 (DOCX 13 kb)

Supplementary file3 (XLSX 18 kb)

## Data Availability

All data generated or analyzed during this study are included in this published article and its supplementary information files.

## References

[1] Fritz S, Lusardi M (2009) White paper:“walking speed: the sixth vital sign.” J Geriatr Phys Ther 32(2):2–5 20039582

[2] Studenski S, Perera S, Patel K, Rosano C, Faulkner K, Inzitari M et al (2011) Gait speed and survival in older adults. JAMA 305(1):50–58 10.1001/jama.2010.1923 PMC3080184 21205966

[3] Rasmussen LJH, Caspi A, Ambler A, Broadbent JM, Cohen HJ, d’Arbeloff T et al (2019) Association of neurocognitive and physical function with gait speed in midlife. JAMA Netw Open 2(10):e1913123 10.1001/jamanetworkopen.2019.13123 PMC6804027 31603488

[4] Middleton A, Fritz SL, Lusardi M (2015) Walking speed: the functional vital sign. J Aging Phys Act 23(2):314–322 10.1123/japa.2013-0236 PMC4254896 24812254

[5] Paluch AE, Bajpai S, Bassett DR, Carnethon MR, Ekelund U, Evenson KR et al (2022) Daily steps and all-cause mortality: a meta-analysis of 15 international cohorts. Lancet Public Heal 7(3):e219–e228 10.1016/S2468-2667(21)00302-9 PMC9289978 35247352

[6] Verghese J, Wang C, Lipton RB, Holtzer R (2013) Motoric cognitive risk syndrome and the risk of dementia. J Gerontol A Biol Sci Med Sci 68(4):412–418 10.1093/gerona/gls191 PMC3593614 22987797

[7] Mirelman A, Bonato P, Camicioli R, Ellis TD, Giladi N, Hamilton JL et al (2019) Gait impairments in Parkinson’s disease. Lancet Neurol 18(7):697–708 10.1016/S1474-4422(19)30044-4 30975519

[8] Churruca K, Pomare C, Ellis LA, Long JC, Henderson SB, Murphy LE et al (2021) Patient‐reported outcome measures (PROMs): a review of generic and condition‐specific measures and a discussion of trends and issues. Health Expect 24(4):1015–1024 10.1111/hex.13254 PMC8369118 33949755

[9] Warmerdam E, Hausdorff JM, Atrsaei A, Zhou Y, Mirelman A, Aminian K et al (2020) Long-term unsupervised mobility assessment in movement disorders. Lancet Neurol 19(5):462–470 10.1016/S1474-4422(19)30397-7 32059811

[10] Erb MK, Karlin DR, Ho BK, Thomas KC, Parisi F, Vergara-Diaz GP et al (2020) Mhealth and wearable technology should replace motor diaries to track motor fluctuations in Parkinson’s disease. NPJ Digit Med 3(1):6 10.1038/s41746-019-0214-x PMC6969057 31970291

[11] Mobbs RJ (2021) From the subjective to the objective era of outcomes analysis: how the tools we use to measure outcomes must change to be reflective of the pathologies we treat in spinal surgery. J Spine Surg 7(3):456 10.21037/jss-2021-2 PMC8511557 34734150

[12] Mobbs RJ, Perring J, Raj SM, Maharaj M, Yoong NKM, Sy LW et al (2022) Gait metrics analysis utilizing single-point inertial measurement units: a systematic review. Mhealth 8:9 10.21037/mhealth-21-17 PMC8800203 35178440

[13] Webster KE, Wittwer JE, Feller JA (2005) Validity of the GAITRite® walkway system for the measurement of averaged and individual step parameters of gait. Gait Posture 22(4):317–321 10.1016/j.gaitpost.2004.10.005 16274913

[14] Pfister A, West AM, Bronner S, Noah JA (2014) Comparative abilities of Microsoft Kinect and Vicon 3D motion capture for gait analysis. J Med Eng Technol 38(5):274–280 10.3109/03091902.2014.909540 24878252

[15] Mercer K, Giangregorio L, Schneider E, Chilana P, Li M, Grindrod K (2016) Acceptance of commercially available wearable activity trackers among adults aged over 50 and with chronic illness: a mixed-methods evaluation. JMIR Mhealth Uhealth. 10.2196/mhealth.4225 PMC4749845 26818775

[16] Hermsen S, Moons J, Kerkhof P, Wiekens C, De Groot M (2017) Determinants for sustained use of an activity tracker: observational study. JMIR Mhealth Uhealth 5(10):e164 10.2196/mhealth.7311 PMC5695980 29084709

[17] Bot BM, Suver C, Neto EC, Kellen M, Klein A, Bare C et al (2016) The mPower study, Parkinson disease mobile data collected using ResearchKit. Sci Data 3(1):160011 10.1038/sdata.2016.11 PMC4776701 26938265

[18] Coravos A, Khozin S, Mandl KD (2019) Developing and adopting safe and effective digital biomarkers to improve patient outcomes. NPJ Digit Med 2(1):14 10.1038/s41746-019-0090-4 PMC6411051 30868107

[19] Tricco AC, Lillie E, Zarin W, O’Brien KK, Colquhoun H, Levac D et al (2018) PRISMA extension for scoping reviews (PRISMA-ScR): checklist and explanation. Ann Intern Med 169(7):467–473 10.7326/M18-0850 30178033

[20] Mollard E, Pedro S, Michaud K (2026) Associations between smartphone‐derived behavioral data and rheumatoid arthritis flares. ACR Open Rheumatol 8(1):e70162 10.1002/acr2.70162 PMC12793784 41521756

[21] Sher A, Rashid M, Lotfi A, Povina F, Akanyeti O (2026) Cycle metrics and strategy detection for automated chair sit-to-stand test analysis employing a single smartphone: A Sher et al. Ann Biomed Eng 54(5):1471–1481 10.1007/s10439-025-03943-4 PMC13091807 41423532

[22] Devito F, Gattulli V, Impedovo D (2026) Care-MOVE: A smartphone-based application for continuous monitoring of mobility, environmental exposure and cognitive status in older patients. Appl Sci 16(3):1549

[23] Schönherr C, Ziegler J, Zentek T, Rashid A, Strauss S, Tallner A et al (2025) Smartphone-based gait analysis in the assessment of fatigue and fatigability in people with multiple sclerosis: a supervised cohort study. J Neurol 272(3):217 10.1007/s00415-025-12906-7 PMC11846754 39985679

[24] Gunter KM, Groenewald K, Aubourg T, Lo C, Welch J, Razzaque J et al (2025) Smartphone-based prediction of dopaminergic deficit in prodromal and manifest Parkinson’s disease. NPJ Digit Med 8:783 10.1038/s41746-025-02148-2 PMC12738540 41326766

[25] Willemse IH, Mellone S, Tacconi C, Ilg W, Schüle R, Synofzik M et al (2025) Smartphone application for spastic ataxias: cross-sectional validation of a newly developed smartphone app for remote monitoring in spastic ataxias. Cerebellum 24(3):71 10.1007/s12311-025-01820-3 PMC11933166 40126682

[26] Durmus K, Bora A, Sapci B, Al-Hazzar MK, Akti K, Kekul Sapci M et al (2025) Diagnostic utility of smartphone-integrated gait analysis in the assessment of BPPV. Front Neurol 16:1728659 10.3389/fneur.2025.1728659 PMC12716158 41426988

[27] Uche SC, Agu E, Grimone K, Herman DS, Abrantes AM, Stein MD (2025) DUI detection from gait using a multichannel 1DCNN-attention-BiLSTM framework. IEEE Access 13:200861–200882 10.1109/access.2025.3636473 PMC12747568 41473808

[28] Kim H-E, Park D-H, An C-H, Choi M-Y, Kim D, Hong Y-S (2025) Real-time detection of distracted walking using smartphone IMU sensors with personalized and emotion-aware modeling. Sensors 25(16):5047 10.3390/s25165047 PMC12390545 40871922

[29] Seo K, Hwang J, Kim K, Argrawal H, Yun JH, Kim HY, editors. End-to-End Classification of Cognitive Impairment Using Daily-Life Gait Data. 2025 47th Annual International Conference of the IEEE Engineering in Medicine and Biology Society (EMBC); 2025: IEEE. 10.1109/EMBC58623.2025.11254035 41336397

[30] Brodke DJ, Shear BM, Demyanovich H, Li V, Bell A, Okhuereigbe D et al (2025) The future is mobile: pilot validation study of Apple Health metrics in orthopaedic trauma. JBJS 107(16):1825 10.2106/JBJS.24.00842 PMC12354133 40833368

[31] Zawada S, Acosta J, Collins C, Dumitrascu O, Harahsheh E, Hagen C et al (2025) Real-world smartphone data predicts mood after ischemic stroke and transient ischemic attack symptoms and may constitute digital endpoints: a proof-of-concept study. Mayo Clin Proc Digit Health 3(3):100240 10.1016/j.mcpdig.2025.100240 PMC12381640 40881107

[32] Suffoletto B, Kim D, Toth C, Mayer W, Ashenburg N, Lin M et al (2025) Development of a model predicting falls in older emergency department patients using smartphone-based mobility measures. J Am Geriatr Soc 73(3):791–801 10.1111/jgs.19303 39653611

[33] Hayek R, Brown RT, Gutman I, Baranes G, Springer S (2025) Smartphone-based analysis for early detection of aging impact on gait and stair negotiation: a cross-sectional study. Sensors (Basel) 25(7):2310 10.3390/s25072310 PMC11991041 40218822

[34] Crowley A, Siegel L, Grainger R, Webster DE, He T, Yang L et al (2025) Clinical validation and outcome measures from bend ease: a novel, sensor-based digital measurement tool for assessing at-home morning stiffness and spinal range of motion in axial spondyloarthritis. Rheumatol Ther 12(2):337–352 10.1007/s40744-025-00746-w PMC11920473 39976661

[35] Powell K, Amer A, Glavcheva-Laleva Z, Williams J, O’Flaherty Farrell C, Harwood F et al (2025) MoveLab®: validation and development of novel cross-platform gait and mobility assessments using gold standard motion capture and clinical standard assessment. Sensors 25(18):5706 10.3390/s25185706 PMC12473320 41012946

[36] Jan K, Alvero AB, Vogel MJ, Wright-Chisem J, Zhu D, Nho SJ (2025) Association of preoperative walking speed with 1-year outcomes after hip arthroscopy for femoroacetabular impingement syndrome. Sports Health 17(6):1279–1286 10.1177/19417381241309918 PMC11748388 39819138

[37] Wang L, Su D, Zhang A, Zhu Y, Jiang W, He X et al (2025) Real-time fall detection using smartphone accelerometers and WiFi channel state information. IEEE Sens J. 10.1109/jsen.2025.3547060

[38] Armoa GJ, Abarca VE, Elias DA, editors 2025. Smartphone-based system for quantifying tremor and freezing of gait in Parkinson’s disease. 10th International Conference on Intelligent Informatics and Biomedical Sciences (ICIIBMS); 2025: IEEE.

[39] Rashid M, Sher A, Povina FV, Akanyeti O (2025) AI-driven adaptive segmentation of timed up and go test phases using a smartphone. Electronics (Basel) 14(23):4650

[40] Farhad M, Choudhury T, Antony BJ, editors 2025. TUG-IoT: A Mobile Integrated IoT based Timed Up and Go Test. Proceedings of the 18th Health Informatics Knowledge Management Conference; 2025.

[41] Shen Y, Benge JF, Rousseau J, Lester-Smith RA, Thomaz E (2025) Passive smartphone sensing reveals gait and typing biomarkers of cognitive impairment. Alzheimers Dement 21:e107368

[42] Tao S, Zhang H, Kong L, Sun Y, Zhao J (2024) Validation of gait analysis using smartphones: reliability and validity. Digit Health 10:20552076241257054 10.1177/20552076241257054 PMC11138199 38817844

[43] Arteaga-Bracho E, Cosne G, Kanzler C, Karatsidis A, Mazzà C, Penalver-Andres J et al (2024) Smartphone-based assessment of mobility and manual dexterity in adult people with spinal muscular atrophy. J Neuromuscul Dis 11(5):1049–1065 10.3233/JND-240004 PMC11380318 38995798

[44] Mejia AC, Gulde P, Salinas CG (2024) A clinical application of gait quality patterns in osteoarthritis. Gait Posture 114:284–289 10.1016/j.gaitpost.2024.10.011 39447427

[45] Ahmad HS, Chauhan D, Dagli MM, Turlip RW, Bashti M, Hamade A et al (2024) Machine learning models leveraging smartphone-based patient mobility data can accurately predict functional outcomes after spine surgery. J Clin Med 13(21):6515 10.3390/jcm13216515 PMC11547021 39518657

[46] Surmacz K, Redfern RE, Van Andel DC, Kamath AF (2024) Machine learning model identifies patient gait speed throughout the episode of care, generating notifications for clinician evaluation. Gait Posture 114:62–68 10.1016/j.gaitpost.2024.09.001 39260073

[47] Nishiyama D, Arita S, Fukui D, Yamanaka M, Yamada H (2024) Accurate fall risk classification in elderly using one gait cycle data and machine learning. Clin Biomech 115:106262 10.1016/j.clinbiomech.2024.106262 38744224

[48] Furtado ECS, Azevedo YSD, Galhardo DdR, Miranda IPC, Oliveira MEC, das Neves PFM et al (2024) Influence of gestational age on pelvic floor muscle activity, plantar contact, and functional mobility in high-risk pregnant women: a cross-sectional study. Sensors (Basel) 24(14):4615 10.3390/s24144615 PMC11280655 39066013

[49] Jin K, Kosa P, Bielekova B (2024) Smartphone tests quantify lower extremities dysfunction in multiple sclerosis. Front Neurol 15:1408224 10.3389/fneur.2024.1408224 PMC11604577 39618842

[50] Nascimento AQ, Nagata LAR, Almeida MT, da Silva Costa VL, de Marin ABR, Tavares VB et al (2024) Smartphone-based inertial measurements during Chester step test as a predictor of length of hospital stay in abdominopelvic cancer postoperative period: a prospective cohort study. World J Surg Oncol 22(1):71 10.1186/s12957-024-03337-1 PMC10900612 38419082

[51] Dorofeev NV, Sharapov RV, Goryachev MS, editors 2024. Predicting health status based on human gait parameters using a smartphone. International Conference on Intelligent Systems;: Springer.

[52] Juneau P, Baddour N, Burger H, Lemaire ED (2024) Balance confidence classification in people with a lower limb amputation using six minute walk test smartphone sensor signals. PLOS Digital Health 3(8):e0000570 10.1371/journal.pdig.0000570 PMC11346636 39186493

[53] Straczkiewicz M, Keating NL, Thompson E, Matulonis UA, Campos SM, Wright AA et al (2023) Open-source, step-counting algorithm for smartphone data collected in clinical and nonclinical settings: algorithm development and validation study. JMIR Cancer 9(1):e47646 10.2196/47646 PMC10687676 37966891

[54] Kocuvan P, Hrastič A, Kareska A, Gams M (2023) Predicting a fall based on gait anomaly detection: A comparative study of wrist-worn three-axis and mobile phone-based accelerometer sensors. Sensors 23(19):8294 10.3390/s23198294 PMC10575458 37837123

[55] Pedrero-Sánchez JF, Belda-Lois JM, Serra-Añó P, Mollà-Casanova S, López-Pascual J (2023) Classification of Parkinson’s disease stages with a two-stage deep neural network. Front Aging Neurosci 15:1152917 10.3389/fnagi.2023.1152917 PMC10272759 37333459

[56] Wall C, Young F, Moore J, McMeekin P, Walker R, Godfrey A, editors 2023. A proposed pervasive smartphone application for personalised gait rehabilitation. 2023 IEEE 19th International Conference on Body Sensor Networks (BSN), IEEE

[57] Shema-Shiratzky S, Beer Y, Mor A, Elbaz A (2022) Smartphone-based inertial sensors technology–validation of a new application to measure spatiotemporal gait metrics. Gait Posture 93:102–106 10.1016/j.gaitpost.2022.01.024 35121485

[58] Omberg L, Chaibub Neto E, Perumal TM, Pratap A, Tediarjo A, Adams J et al (2022) Remote smartphone monitoring of Parkinson’s disease and individual response to therapy. Nat Biotechnol 40(4):480–487 10.1038/s41587-021-00974-9 PMC12812035 34373643

[59] Juneau P, Baddour N, Burger H, Bavec A, Lemaire ED (2022) Amputee fall risk classification using machine learning and smartphone sensor data from 2-minute and 6-minute walk tests. Sensors (Basel) 22(5):1749 10.3390/s22051749 PMC8914626 35270892

[60] Alexander S, Braisher M, Tur C, Chataway J (2022) The mSteps pilot study: analysis of the distance walked using a novel smartphone application in multiple sclerosis. Mult Scler J 28(14):2285–2293 10.1177/13524585221124043 36177917

[61] Ellis R, Kelly P, Huang C, Pearlmutter A, Izmailova ES (2022) Sensor verification and analytical validation of algorithms to measure gait and balance and pronation/supination in healthy volunteers. Sensors 22(16):6275 10.3390/s22166275 PMC9412295 36016036

[62] Thakur D, Biswas S (2022) Attention-based deep learning framework for hemiplegic gait prediction with smartphone sensors. IEEE Sens J 22(12):11979–11988

[63] Pedrero-Sánchez JF, Belda-Lois J-M, Serra-Ano P, Ingles M, Lopez-Pascual J (2022) Classification of healthy, Alzheimer and Parkinson populations with a multi-branch neural network. Biomed Signal Process Control 75:103617

[64] Juneau P, Lemaire ED, Bavec A, Burger H, Baddour N (2022) Automated step detection with 6-minute walk test smartphone sensors signals for fall risk classification in lower limb amputees. PLOS Digit Heal 1(8):e0000088 10.1371/journal.pdig.0000088 PMC9931302 36812591

[65] Su D, Liu Z, Jiang X, Zhang F, Yu W, Ma H et al (2021) Simple smartphone-based assessment of gait characteristics in Parkinson disease: validation study. JMIR Mhealth Uhealth 9(2):e25451 10.2196/25451 PMC7935653 33605894

[66] Soangra R, Lockhart T (2021) Smartphone-based prediction model for postoperative cardiac surgery outcomes using preoperative gait and posture measures. Sensors 21(5):1704 10.3390/s21051704 PMC7958120 33801240

[67] Alberto S, Cabral S, Proença J, Pona-Ferreira F, Leitão M, Bouça-Machado R et al (2021) Validation of quantitative gait analysis systems for Parkinson’s disease for use in supervised and unsupervised environments. BMC Neurol 21(1):331 10.1186/s12883-021-02354-x PMC8403450 34454453

[68] Van Oirschot P, Heerings M, Wendrich K, Den Teuling B, Dorssers F, Van Ee R et al (2021) A two-minute walking test with a smartphone app for persons with multiple sclerosis: validation study. JMIR Form Res 5(11):e29128 10.2196/29128 PMC8663688 34787581

[69] de Carvalho LR, de Paula AR, Silva AFS, Costa PHV, Polese JC (2021) Validity of mHealth devices for counting steps in individuals with Parkinson’s disease. J Bodyw Mov Ther 28:496–501 10.1016/j.jbmt.2021.06.018 34776185

[70] Shahar RT, Agmon M (2021) Gait analysis using accelerometry data from a single smartphone: agreement and consistency between a smartphone application and gold-standard gait analysis system. Sensors 21(22):7497 10.3390/s21227497 PMC8622042 34833576

[71] Patel B, Srikanthan S, Asani F, Agu E, editors 2021. Machine learning prediction of tbi from mobility, gait and balance patterns. 2021 IEEE/ACM Conference on Connected Health: Applications, Systems and Engineering Technologies (CHASE). IEEE

[72] Zhai Y, Nasseri N, Pöttgen J, Gezhelbash E, Heesen C, Stellmann J-P (2020) Smartphone accelerometry: A smart and reliable measurement of real-life physical activity in multiple sclerosis and healthy individuals. Front Neurol 11:688 10.3389/fneur.2020.00688 PMC7456810 32922346

[73] Abujrida H, Agu E, Pahlavan K (2020) Machine learning-based motor assessment of Parkinson’s disease using postural sway, gait and lifestyle features on crowdsourced smartphone data. Biomed Phys Eng Exp 6(3):035005 10.1088/2057-1976/ab39a8 33438650

[74] Suffoletto B, Dasgupta P, Uymatiao R, Huber J, Flickinger K, Sejdic E (2020) A preliminary study using smartphone accelerometers to sense gait impairments due to alcohol intoxication. J Stud Alcohol Drugs 81(4):505–510 10.15288/jsad.2020.81.505 PMC7437548 32800088

[75] Motolese F, Magliozzi A, Puttini F, Rossi M, Capone F, Karlinski K et al (2020) Parkinson’s disease remote patient monitoring during the COVID-19 lockdown. Front Neurol 11:567413 10.3389/fneur.2020.567413 PMC7575750 33117262

[76] Borzì L, Olmo G, Artusi CA, Lopiano L, editors 2020. Detection of freezing of gait in people with Parkinson’s disease using smartphones. 2020 IEEE 44th Annual Computers, Software, and Applications Conference (COMPSAC). IEEE

[77] Hashmi MA, Riaz Q, Zeeshan M, Shahzad M, Fraz MM (2020) Motion reveal emotions: Identifying emotions from human walk using chest mounted smartphone. IEEE Sens J 20(22):13511–13522

[78] Nutakki C, Mathew RJ, Suresh A, Vijay AR, Krishna S, Babu AS et al (2020) Classification and kinetic analysis of healthy gait using multiple accelerometer sensors. Procedia Comput Sci 171:395–402

[79] Sarda A, Munuswamy S, Sarda S, Subramanian V (2019) Using passive smartphone sensing for improved risk stratification of patients with depression and diabetes: cross-sectional observational study. JMIR Mhealth Uhealth 7(1):e11041 10.2196/11041 PMC6371066 30694197

[80] Gerhardy T, Gordt K, Jansen C-P, Schwenk M (2019) Towards using the instrumented timed up-and-go test for screening of sensory system performance for balance control in older adults. Sensors (Basel) 19(3):622 10.3390/s19030622 PMC6387355 30717202

[81] Lo C, Arora S, Baig F, Lawton MA, El Mouden C, Barber TR et al (2019) Predicting motor, cognitive & functional impairment in Parkinson’s. Annals Clin Transl Neurol 6(8):1498–1509 10.1002/acn3.50853 PMC6689691 31402628

[82] Chomiak T, Xian W, Pei Z, Hu B (2019) A novel single-sensor-based method for the detection of gait-cycle breakdown and freezing of gait in Parkinson’s disease: T. Chomiak et al. J Neural Transm 126(8):1029–1036 10.1007/s00702-019-02020-0 31154512

[83] Ramkumar PN, Haeberle HS, Ramanathan D, Cantrell WA, Navarro SM, Mont MA et al (2019) Remote patient monitoring using mobile health for total knee arthroplasty: validation of a wearable and machine learning–based surveillance platform. J Arthroplasty 34(10):2253–2259 10.1016/j.arth.2019.05.021 31128890

[84] Kim HB, Lee HJ, Lee WW, Kim SK, Jeon HS, Park HY et al (2018) Validation of freezing-of-gait monitoring using smartphone. Telemed e-Health 24(11):899–907 10.1089/tmj.2017.0215 29708870

[85] Hemmatpour M, Ferrero R, Gandino F, Montrucchio B, Rebaudengo M (2018) Nonlinear predictive threshold model for real-time abnormal gait detection. J Healthc Eng 2018(1):4750104 10.1155/2018/4750104 PMC6038668 30046416

[86] Suffoletto B, Gharani P, Chung T, Karimi H (2018) Using phone sensors and an artificial neural network to detect gait changes during drinking episodes in the natural environment. Gait Posture 60:116–121 10.1016/j.gaitpost.2017.11.019 PMC5809199 29179052

[87] Hurt CP, Lein DH Jr, Smith CR, Curtis JR, Westfall AO, Cortis J et al (2018) Assessing a novel way to measure step count while walking using a custom mobile phone application. PLoS ONE 13(11):e0206828 10.1371/journal.pone.0206828 PMC6219786 30399162

[88] Soangra R, Lockhart TE (2018) Inertial sensor-based variables are indicators of frailty and adverse post-operative outcomes in cardiovascular disease patients. Sensors (Basel) 18(6):1792 10.3390/s18061792 PMC6021795 29865245

[89] Ebara T, Azuma R, Shoji N, Matsukawa T, Yamada Y, Akiyama T et al (2017) Reliability of smartphone-based gait measurements for quantification of physical activity/inactivity levels. J Occup Heal 59(6):506–512 10.1539/joh.17-0101-OA PMC5721272 28835575

[90] Low CA, Dey AK, Ferreira D, Kamarck T, Sun W, Bae S et al (2017) Estimation of symptom severity during chemotherapy from passively sensed data: exploratory study. J Med Internet Res 19(12):e420 10.2196/jmir.9046 PMC5750420 29258977

[91] Sasidhar K, Satyajeet S, editors 2017. iKnow how you walk—A smartphone based personalized gait diagnosing system. 2017 International Conference on Advances in Computing, Communications and Informatics (ICACCI). IEEE

[92] Raknim P, Lan K-c (2016) Gait monitoring for early neurological disorder detection using sensors in a smartphone: validation and a case study of parkinsonism. Telemed J E Health 22(1):75–81 10.1089/tmj.2015.0005 26302109

[93] Wang C, Lee S, Ho J, Na Y, Min SD, editors 2016. Detection of optimal activity recognition algorithm for elderly using smartphone. International Conference on Computer Science and its Applications. Springer. Newyork

[94] Chan MH, Keung DT, Lui SY, Cheung RT (2016) A validation study of a smartphone application for functional mobility assessment of the elderly. Hong Kong Physiother J 35:1–4 10.1016/j.hkpj.2015.11.001 PMC6385142 30931027

[95] Furrer M, Bichsel L, Niederer M, Baur H, Schmid S (2015) Validation of a smartphone-based measurement tool for the quantification of level walking. Gait Posture 42(3):289–294 10.1016/j.gaitpost.2015.06.003 26141906

[96] Arnold Z, Larose D, Agu E, editors 2015. Smartphone inference of alcohol consumption levels from gait. 2015 International Conference on Healthcare Informatics. IEEE

[97] Lan K-C, Shih W-Y, editors 2015. Early detection of neurological disease using a smartphone: a case study. 2015 9th international conference on sensing technology (ICST). IEEE

[98] Nishiguchi S, Ito H, Yamada M, Yoshitomi H, Furu M, Ito T et al (2014) Self-assessment tool of disease activity of rheumatoid arthritis by using a smartphone application. Telemed e-Health 20(3):235–240 10.1089/tmj.2013.0162 24404820

[99] Kolber MJ, Pizzini M, Robinson A, Yanez D, Hanney WJ (2013) The reliability and concurrent validity of measurements used to quantify lumbar spine mobility: an analysis of an iphone® application and gravity based inclinometry. Int J Sports Phys Ther 8(2):129 PMC3625792 23593551

[100] Cheng Q, Juen J, Li Y, Prieto-Centurion V, Krishnan JA, Schatz BR, editors 2013. GaitTrack: Health monitoring of body motion from spatio-temporal parameters of simple smart phones. Proceedings of the International Conference on Bioinformatics, Computational Biology and Biomedical Informatics

[101] How T-V, Chee J, Wan E, Mihailidis A, editors 2013. MyWalk: a mobile app for gait asymmetry rehabilitation in the community. 2013 7th International Conference on Pervasive Computing Technologies for Healthcare and Workshops. IEEE

[102] Majumder AJA, Rahman F, Zerin I, Ebel Jr W, Ahamed SI, editors 2013. iPrevention: towards a novel real-time smartphone-based fall prevention system. Proceedings of the 28th Annual ACM Symposium on Applied Computing

[103] Noury N, Quach K-A, Berenguer M, Bouzi M-J, Teyssier H, editors 2012. A feasibility study of using a smartphone to monitor mobility in elderly. 2012 IEEE 14th International Conference on e-Health Networking, Applications and Services (Healthcom). IEEE

[104] Rochester L, Mazzà C, Mueller A, Caulfield B, McCarthy M, Becker C et al (2020) A roadmap to inform development, validation and approval of digital mobility outcomes: the Mobilise-D approach. Digital Biomark 4(Suppl. 1):13–27 10.1159/000512513 PMC7768123 33442578

[105] Apple Inc. (2021) Measuring walking quality through iPhone mobility metrics. Apple Inc., Cupertino.

[106] Mobbs RJ, Mobbs RR, Choy WJ (2019) Proposed objective scoring algorithm for assessment and intervention recovery following surgery for lumbar spinal stenosis based on relevant gait metrics from wearable devices: the gait posture index (GPi). J Spine Surg 5(3):300 10.21037/jss.2019.09.06 PMC6787370 31663040

[107] Hausdorff JM (2005) Gait variability: methods, modeling and meaning. J Neuroeng Rehabil 2(1):19 10.1186/1743-0003-2-19 PMC1185560 16033650

[108] Kalla R, Tiller N, Goyal M, Brémovà-Ertl T (2026) Gait as a vital sign: integrating wearables and AI into vestibular and balance medicine. Front Neurol 17:1736898 10.3389/fneur.2026.1736898 PMC12971410 41815726

