# Supplementary material for: Clinical utility and methodological implementation of smartphone-based mobility analysis: a scoping review
Source: J Neurol. 2026 Sep 15;273(10):597. doi: 10.1007/s00415-026-14131-2 (PMC13577997; doi:10.1007/s00415-026-14131-2)
Supplement: Supplementary file 2 [file 415_2026_14131_MOESM2_ESM.docx]

(Smartphone*[tiab] OR iPhone*[tiab] OR Android[tiab] OR "Mobile phone*"[tiab] OR "Apple Health"[tiab] OR "Google Fit"[tiab]) AND

(Gait[tiab] OR Mobility[tiab]) AND

("Clinical utility"[tiab] OR Triage[tiab] OR Validation[tiab] OR "Early detection"[tiab] OR Predictive[tiab])

(Smartphone* OR iPhone* OR Android OR "Mobile phone*" OR "Apple Health" OR "Google Fit").ti,ab. AND

(Gait OR Mobility).ti,ab. AND

("Clinical utility" OR Triage OR Validation OR "Early detection" OR Predictive).ti,ab.

TITLE-ABS((Smartphone* OR iPhone* OR Android OR "Mobile phone*" OR "Apple Health" OR "Google Fit") AND

(Gait OR Mobility) AND

("Clinical utility" OR Triage OR Validation OR "Early detection" OR Predictive))
